# Prognostic and Clinical Significance of PD-L1, EGFR and Androgen Receptor (AR) Expression in Triple-Negative Breast Cancer (TNBC) Patients

**DOI:** 10.3390/life14060682

**Published:** 2024-05-26

**Authors:** Nataša Medić-Milijić, Irena Jovanić, Milica Nedeljković, Ivan Marković, Igor Spurnić, Zorka Milovanović, Nejla Ademović, Tijana Tomić, Nasta Tanić, Nikola Tanić

**Affiliations:** 1 Department of Pathology, Institute of Oncology and Radiology of Serbia, 11000 Belgrade, Serbia; natasamm76@gmail.com (N.M.-M.); jovanicirena@gmail.com (I.J.); zmilovanovic@ncrc.ac.rs (Z.M.); 2 Department of Experimental Oncology, Institute of Oncology and Radiology of Serbia, 11000 Belgrade, Serbia; mnedel30@tutanota.com; 3 Surgical Oncology Clinic, Institute of Oncology and Radiology of Serbia, 11000 Belgrade, Serbia; ivanmarkovic66@yahoo.com (I.M.); spurnic@gmail.com (I.S.); 4 Department of Neurobiology, Institute for Biological Research “Siniša Stanković”, National Institute of Republic of Serbia, University of Belgrade, 11060 Belgrade, Serbia; nejla.ademovic@ibiss.bg.ac.rs; 5 Department of Radiobiology and Molecular Genetics, Institute of Nuclear Sciences “Vinča”, National Institute of Republic of Serbia, University of Belgrade, Mike Petrocića Alasa 12-14, 11000 Belgrade, Serbia; tijana.tomic@vin.bg.ac.rs (T.T.); nastad@vinca.rs (N.T.); 6 Department of Natural Sciences and Mathematics, Field of Biology, State University of Novi Pazar, 36300 Novi Pazar, Serbia

**Keywords:** triple-negative breast cancer (TNBC), PD-L1, AR, EGFR, Ki67

## Abstract

**Simple Summary:**

Breast cancer is the most commonly occurring malignancy and the leading cause of cancer-related death in women. The most aggressive breast cancer subtype is triple-negative breast cancer (TNBC). It is associated with high recurrence rates, a high incidence of distant metastases and poor overall survival. The aim of this study was to investigate the role of PD-L1 (programmed death-ligand 1), EGFR (epidermal growth factor receptor) and androgen receptor (AR) expression in TNBC promotion and progression. To that end, we analyzed the immunoexpression of these genes in 125 postoperative samples of TNBC patients and correlated the obtained results with clinicopathological parameters and survival. According to the obtained results, we believe that a concurrent examination of PD-L1, AR, EGFR and Ki67 (in addition) protein expression may be more useful in predicting TNBC clinical course than the analysis of the individual expression of any of these proteins. Specifically, our results showed that a simultaneous low expression of PD-L1, low expression of AR and high expression of EGFR, followed by an elevated expression of Ki67, constitute a ‘high risk’ profile of TNBC. If we combine these results with our previous findings, which showed that reduced PTEN/high PI3K/high mTOR expression represents an unfavorable condition, we could have a promising formula for better prediction of TNBC outcome and a better starting point for new treatment modalities.

**Abstract:**

Triple-negative breast cancer (TNBC) is the most aggressive breast cancer subtype and is associated with high recurrence rates, a high incidence of distant metastases and poor overall survival. The aim of this study was to investigate the role of PD-L1, EGFR and AR expression in TNBC promotion and progression. To that end, we analyzed the immunohistochemical expression of these genes in 125 TNBC patients and their relation to clinicopathological parameters and survival. An elevated expression of PD-L1 was significantly correlated with higher tumor and nuclear grade, while a low expression was correlated with loco-regional recurrence without any influence on survival. Contrary to this, the expression of AR showed a positive impact on the DFI and a negative association with tumor grade. Furthermore, PD-L1 and AR demonstrated simultaneous expression, and further co-expression analysis revealed that a positive expression of PD-L1/AR notably correlates with tumor and nuclear grade and has a significant impact on a longer DFI and OS, while a negative PD-L1/AR expression is significantly associated with metastases. Therefore, our results suggest that positive PD-L1/AR expression is beneficial for TNBC patients. In addition, an elevated expression of EGFR contributes to metastases and a worse DFI and OS. In conclusion, we think that low PD-L1/low AR/high EGFR expression followed by high Ki67 expression constitutes a ‘high risk’ profile of TNBC.

## 1. Introduction

Breast cancer (BC) is the most commonly occurring malignancy and the leading cause of cancer-related death among women worldwide [1]. Breast cancer is an exceptionally heterogeneous group of malignant diseases and is generally classified as (a) hormone-dependent (ER+), which expresses steroid receptors (estrogen receptor—ER, progesterone receptor—PR) but has a variable human epidermal growth factor receptor 2 (HER2) expression, and (b) triple-negative breast cancers (TNBCs), which do not express either of them. TNBC represents 10–15% of all breast cancers and is a relatively small entity when compared to hormone-positive tumors, which represent 80% but are the most aggressive breast cancer subtype [2]. Namely, TNBC is associated with high recurrence rates, a high incidence of distant metastases and poor overall survival [3,4]. Moreover, TNBC is characterized by a lack of solid biomarkers and the absence of any response to currently available targeted therapies, while systemic treatment options are limited only to cytotoxic drugs. However, despite better initial response rates, the majority of TNBCs develop resistance to chemotherapy [5]. Therefore, defining better molecular markers for better TNBC subtype stratification and clarifying the mechanisms of drug resistance are of crucial importance for their successful therapy treatment.

TNBC can be determined by morphological and IHC (immunohistochemistry) profiling. IHC analysis, particularly the expression analyses of steroid receptors (ER, PR and HER2), is essential for TNBC phenotyping [6]. Simultaneous IHC analysis of Ki-67 is of additional importance, since the level of Ki-67 expression is a marker of proliferation and is considered to be a prognostic biomarker of disease-free survival (DFS) and overall survival (OS) in breast cancer patients, especially TNBC [7].

Another promising, potentially very important, IHC marker for TNBC patients is a member of the steroid receptor superfamily, i.e., androgen receptor (AR). AR is a ligand-dependent transcription factor that is expressed in many human tissues. It is expressed in TNBC, with the expression level between 10 and 90% according to the cohort and the cutoff used for AR positivity [8,9]. Although there have been several reviews discussing the role of AR, its function appears to vary among the diverse breast cancer subtypes. In other words, its prognostic and predictive value in BC patients remains controversial [10].

Another receptor, important for BC promotion and progression, is epidermal growth factor receptor (EGFR), which plays an important role in cell proliferation and apoptosis inhibition. EGFR overexpression in TNBC is variable among studies, ranging from 13 to 78% [11]. It looks like EGFR is responsible, among other things, for the maintenance of AR signaling in hormone-depleted environments, i.e., AR can be activated by EGFR [12]. However, some studies showed that AR controls EGFR gene expression [13]. So, it seems like there is a fine interplay and correlation between them [14]. Furthermore, EGFR is involved in the regulation of programmed death-ligand 1 (PD-L1) expression and cell proliferation via the IL-6/JAK/STAT3 signaling pathway in some cancers (for example, non-small-cell lung cancer). PD-L1 is a transmembrane protein and is the ligand of programmed cell death-1 (PD-1), and it is expressed in various cancers (including some TNBCs) and tumor-infiltrating inflammatory and immune cells. Once PD-L1 binds to PD-1, found in activated T-cells and B-cells, it inhibits T-cell migration and proliferation and the secretion of cytotoxic mediators, thereby limiting their killing effect on tumor cells and playing important pathogenic role in cancer progression [15]. Traditionally, breast cancer has not been considered as immunogenic tumor. However, recent developments have shown that the most aggressive BC type, (TNBC), is immunogenic [16], so that immune checkpoint molecules from the PD-1/PD-L1 pathway are considered good future targets for immune therapy.

The aim of this study was to investigate the role of PD-L1, EGFR and AR expression in TNBC promotion, progression and response to therapy. More precisely, we aimed to investigate the impact of the expression of these genes individually and as a package of genes on TNBC and to discuss the obtained results with our previous findings on another package of genes, which showed reduced PTEN/high PI3K/high mTOR expression as a “high risk” TNBC profile.

## 2. Materials and Methods

### 2.1. Patients and Tissue Samples

**Figure 1:**
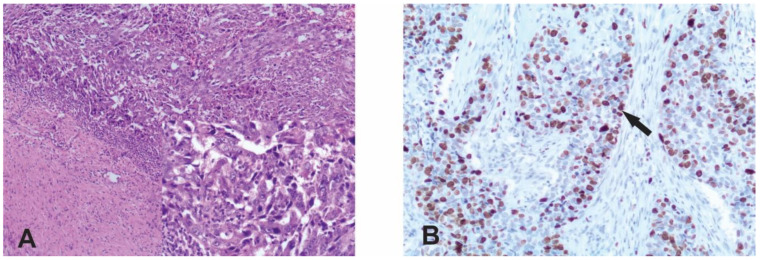
Triple-negative breast cancer. (**A**) H&E staining ×40. In the lower right corner, a segment of the same preparation is enlarged (×100) in order to better see the morphology of tumor cells; (**B**) high level of KI-67 nuclear immunohistochemical staining. Arrow shows a stained nucleus. Magnification ×100.

This was a retrospective study that included 125 patients with early and locally advanced triple-negative breast cancer who were surgically treated and resected at the Institute of Oncology and Radiology of Serbia in Belgrade in the period from 2009 to 2014. The follow-up period was from the date of surgery to the end of 2022. All examined tumor samples were formalin-fixed and paraffin-embedded. Histopathological diagnosis of the primary tumor was carried out according to standard hematoxylin and eosin (H&E)-stained serial sections after radical mastectomy or partial breast resection or subcutaneous mastectomy with ipsilateral axillary node dissection (Figure 1A). Histological type, histological grade, nuclear grade, tumor size and the status of the axillary lymph nodes were determined. Tumors were classified according to the histological classification of the World Health Organization (WHO) [17] and graded according to the Nottingham grading system (Nottingham grading system) [18]. The pathological stage was determined according to the pTNM classification [19]. ER, PR and HER/2 statuses were routinely assessed using semi-quantitative commercial IHC assays according to the manufacturers’ recommendations (monoclonal antibodies: for ER, Clone EP1, Dako; Dako Autostainer Link 48; for PR, Clone PgR636, Dako; Dako Autostainer Link 48; and for HER-2, Clone 4B5, Ventana; Ventana Bench Mark-GX). The detection and visualization system for ER and PR was an En Vision FLEX, Hihg pH and Ultra View Universal DAB Detection Kit for HER-2. Detailed clinical records for the patients included in this study are summarized in Table 1. Approval for this study was obtained from the Ethics Committee of the Institute for Oncology and Radiology of Serbia, number 4321-01. All samples were collected and analyzed as per the ethical standards laid down in the 1964 Declaration of Helsinki.

### 2.2. Immunohistochemistry

The expression status of ER, PR, HER-2, Ki-67, AR, EGFR and PD-L1 was analyzed by the immunohistochemical staining method (IHC). IHC staining was performed on 4 μm sections of formalin-fixed, paraffin-embedded tissues. The automated IHC method was used to detect the expression of ER, PR, HER-2, Ki-67, AR and PD-L1. The following monoclonal antibodies were used: Clone 30-9, Ventana; Ventana Bench Mark-GX for Ki-67, Clone SP107, Cell Marque; Ventana Bench Mark-GX for AR and Clone 22C3, Dako; Dako Autostainer Link 48 for PD-L1 (monoclonal antibodies used for detection of ER, PR and HER-2 are listed in the above paragraph). The system for the detection and visualization of HER-2, Ki-67 and AR was an Ultra View Universal DAB Detection Kit and PD-L1 IHC 22C3 Pharm DX for PD-L1.

The manual IHC method was used to detect the expression of EGFR. Tissue sections were deparaffinized and rehydrated in graded alcohols followed by distilled water and then incubated in 3% hydrogen peroxide for 10 min. Slides were immersed in pH 8 buffer and heated in a microwave at 95 °C for 29 min for antigen retrieval. They were left to cool down at room temperature for 10 min and rinsed with phosphate-buffered saline followed by primary monoclonal antibody (EGFR.113, Novocastra, 1:14). The system used for detection visualization was an UltraVision Quanto Detection System HRP + DAB Quanto (Epredia).

### 2.3. Evaluation of Staining

**Figure 2:**
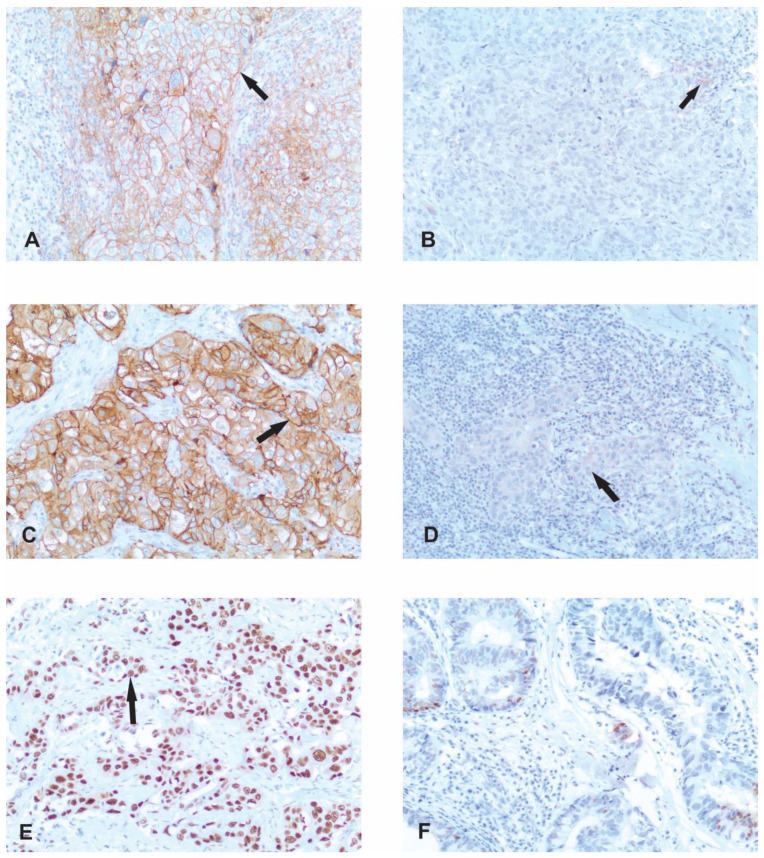
Immunohistochemical staining of PD-L1, EGFR and AR in triple-negative breast cancer. (**A**) Positive result of PD-L1 immunohistochemical staining with CPS 95. Membrane staining of a tumor cell is shown (indicated by an arrow). Magnification ×100. (**B**) Negative result of PD-L1 immunohistochemical staining with CPS below 10. Arrow points to a rare positive stained immune cell. Magnification ×100. (**C**) Positive result of EGFR immunohistochemical staining with Allred score of 8. Strong membrane staining of most tumor cells is visible (pointed by an arrow). Magnification ×100. (**D**) Negative result of EGFR immunohistochemical staining with Allred score of 2. The arrow points to weak membrane staining of some tumor cells. Magnification ×100. (**E**) Positive result of AR nuclear immunohistochemical staining in 95% of tumor cells. Stained nucleus is showcased (pointed by an arrow). Magnification ×100. (**F**) Negative result of AR immunohistochemical staining of less than 10% of stained tumor cells. Magnification ×100.

Staining was assessed independently by two pathologists. The expression status of ER, PR and EGFR was determined by the Allred scoring system, which is based on the sum of the scores of the percentage of stained nuclei of malignant cells (0—no immunoreactivity, 1—<1% of stained nuclei, 2—1–10% of stained nuclei, 3—11–33% of stained nuclei, 4—34–66% of stained nuclei and 5—67–100% of stained nuclei) and staining intensity (0—no immunoreactivity, 1—weak staining intensity, 2—moderate staining intensity and 3—strong staining intensity) [20,21]. Negative cases for ER and PR were considered those with a score < 3 [22]. For EGFR, a score ≥ 4 was considered positive [21] (Figure 2C,D).

Evaluation of HER-2 immunoreactivity was carried out based on the assessment of membrane staining, i.e., tumor cell intensity and continuity of staining. A score of 0 indicated no staining or weak incomplete staining in ≤10% of invasive tumor cells, a score of 1+ indicated weak incomplete staining in >10% of invasive tumor cells, a score of 2+ indicated weak-to-moderate complete staining in >10% of invasive tumor cells or strong complete and circumferential staining in ≤10% of invasive tumor cells and a score of 3+ indicated strong complete and circumferential staining in >10% of invasive tumor cells. HER-2 2+ tumors were retested by the CISH, Dual-CISH or Dual-SISH methods for the assessment of HER2 amplification. [23,24]

The scoring system for Ki-67 is defined as the percentage of stained nuclei of malignant cells of any intensity. The Ki-67 expression level was defined as low (≤15%), intermediate (16–30%) and high (>30%) [25] (Figure 1B).

The expression status of AR was determined by a quantitative method based on the percentage of nuclear expression of malignant cells of any intensity. The cut-off value used for positive expression was ≥10% [26] (Figure 2E,F).

Immunoreactivity of viable tumor cells, lymphocytes and macrophages was examined in order to analyze the PD-L1 status. Partial or complete linear membrane staining of any intensity in malignant cells and any membrane and/or cytoplasmic staining of any intensity for lymphocytes and macrophages were scored. Only lymphocytes and macrophages directly related to the tumor tissue were included. The CPS score is presented as PD-L1 staining cells (tumor cells, lymphocytes, macrophages) divided by the total number of viable tumor cells multiplied by 100. The CPS score was evaluated at a magnification of 20×. A positive CPS score was ≥10, while a negative score was <10 [27] (Figure 2A,B).

### 2.4. Statistical Analysis

Data analysis was performed using the GraphPad Prism 8 software (GraphPad Software, Inc., San Diego, CA, USA) and IBM SPSS Statistics version 25 for Windows (IBM Corporation, Armonk, NY, USA). Correlations between the expressions of the analyzed proteins (Ki-67, AR, EGFR and PD-L1) in TNBC were examined using Spearman’s rank test. Associations of clinicopathological parameters of TNBC patients with the immunoexpression status of the analyzed proteins were determined using Fisher’s exact test, Fisher’s exact test with the Freeman–Halton extension or the Chi-square test, depending on the test conditions. Survival distributions were estimated by the Kaplan–Meier product-limit method, and the log-rank test was used to determine the significance of the differences between survival curves. The disease-free interval (DFI) was calculated from the day after surgery to the first day of disease progression, while overall survival (OS) was calculated from the day after surgery to the last follow-up examination or death of the patient. All performed statistical tests were two-tailed. Statistical differences were considered significant for *p* ≤ 0.05.

## 3. Results

Our study included 125 triple-negative breast cancer patients. The levels and cellular distribution of protein expressions of PD-L1, EGFR and AR were analyzed by immunohistochemistry (Figure 2). An elevated expression of PD-L1 was detected in 50 cases (40%) out of 125. Immunoreactivity was exclusively present in the membrane of malignant cells (Figure 2A) and occasionally in the membrane of tumor-infiltrating inflammatory and immune cells (Figure 2B). The same pattern of expression, with membrane immunoreactivity, was detected for EGFR (Figure 2C). A high level of immunoexpression was obtained in 42 patients (34%), while the rest of the patients demonstrated no expression or weak membrane staining of some tumor cells (Figure 2D). Contrary to PD-L1 and EGFR, AR displayed nuclear expression. A high expression of AR was observed in 46 cases (37%) of the analyzed cohort. It is worth mentioning that a positive expression of AR was observed in almost 95% of the tumor cells (Figure 2E) of these patients, while no expression was seen in less than 10% of the tumor cells (Figure 2F).

### Clinical Significance of PD-L1, AR and EGFR Expression in TNBC Patients

**Figure 3:**
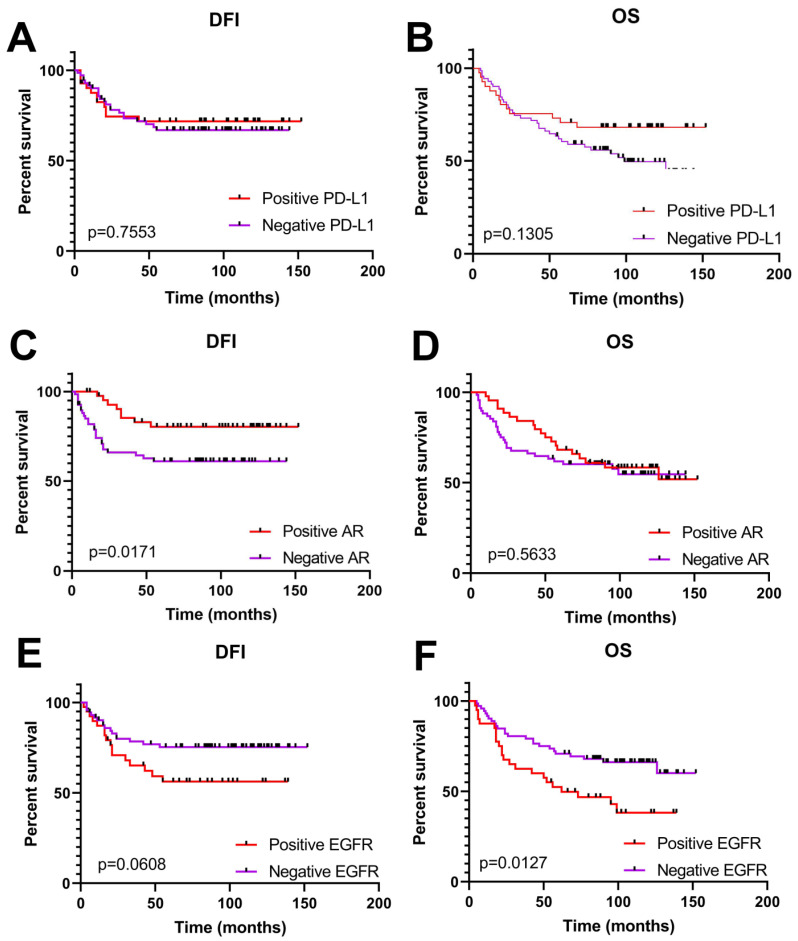
Kaplan–Meier survival curves according to the immunoexpression of PD-L1, AR and EGFR genes in the TNBC cohort group. DFI, disease-free interval; OS, overall survival. (**A**,**B**) Elevated immunoexpression of PD-L1 did not affect disease-free interval and overall survival of patients. (**C**) Patients with elevated immunoexpression of AR had significantly longer DFI. (**D**) Expression of AR did not affect patient OS. (**E**,**F**) Patients with high EGFR expression had significantly shorter OS with no significant influence on DFI.

The relationship between PD-L1, AR and EGFR expression and clinicopathological parameters of TNBC in the examined cohort are summarized in Table 2. Our results showed that an elevated expression of PD-L1 was significantly correlated with a higher tumor grade (*p* = 0.0002) and nuclear grade (*p* = 0.0007) of TNBCs. Unexpectedly and controversially to these findings, a negative PD-L1 expression was associated with loco-regional recurrence (*p* = 0.0146). No correlation was found with the other clinicopathological parameters. In addition, the expression of PD-L1 did not show any significant influence on survival, the disease-free interval (DFI) or overall survival (OS) (Figure 3A,B). Contrary to this finding, the expression of AR affected survival, specifically the DFI (disease-free interval, *p* = 0.0171), but not OS (Figure 3C,D). In effect, a positive AR expression was associated with a longer DFI. In other words, a positive expression of AR demonstrated beneficial effects. In accordance with this finding, an elevated AR expression was negatively correlated with tumor grade (*p* = 0.0491), i.e., a positive expression was associated with a lower tumor grade (Table 2). Once again, a positive AR expression demonstrated beneficial effects, while a negative expression was associated with bad effects.

**Figure 4:**
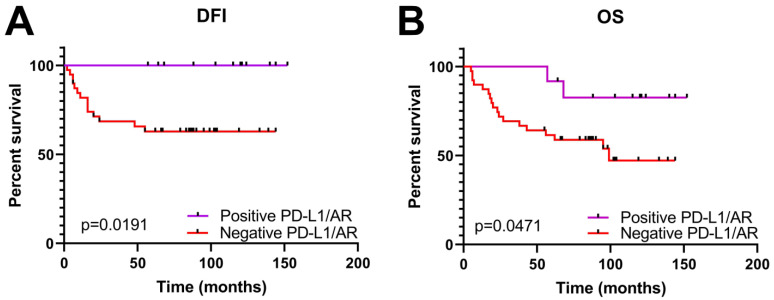
Kaplan–Meier survival curves according to simultaneous expression of PD-L1 and AR proteins in TNBC cohort. Positive PD-L1/AR when both were expressed; negative PD-L1/AR when there was lack of their expression; DFI disease-free interval; OS, overall survival. (**A**) Patients with high expression of PD-L1 and AR had a significantly longer DFI. (**B**) Patients with high expression of PD-L1 and AR had a significantly longer overall survival (OS).

Further analysis revealed that the expression of PD-L1 and AR were in significant correlation (Spearman’s r −0.2747; 95% confidence interval −0.4339 to −0.09895: *p* (two-tailed) 0.0019). This result enabled us to divide the patients into two distinct groups, i.e., those who had a positive simultaneous expression of both genes and those who did not, and to analyze them in pairs. Our analysis revealed that a simultaneous expression of PD-L1 and AR was significantly positively correlated with TNBC tumor grade (*p* = 0.05) and nuclear grade (*p* = 0.0242). On the other hand, a negative expression of both proteins was associated with metastatic events (*p* = 0.0497) (Table 3). In addition, a concurrent expression of PD-L1 and AR demonstrated a significant impact on survival, the DFI (*p* = 0.0191) and OS (*p* = 0.0471). Patients with a concurrent positive expression of PD-L1 and AR lived longer; they had both a longer DFI and OS (Figure 4).

It is important to emphasize that the expression of Ki67, the marker of proliferation, was absolutely correlated with the expression of PD-L1 and AR. In other words, the expression of Ki67 was positively correlated with the expression of PD-L1 and was negatively with the expression of AR (Table 2). These results imply that the pattern of Ki67 expression could support the idea that PD-L1 expression is bad news for TNBC patients, while AR expression might be a good development.

In addition, we analyzed the expression status of EGFR. Not surprisingly, our study revealed that an elevated expression of EGFR contributes to metastases (*p* = 0.0249) (Table 2). Moreover, a high expression of EGFR has a significant impact (*p* = 0.0127) on the poor overall survival of patients (Figure 3E,F).

## 4. Discussion

The main purpose of this study was to analyze the expression of the PD-L1 (programmed death-ligand 1), androgen receptor (AR) and EGFR (epidermal growth factor receptor) proteins in TNBC patients and their role in TNBC promotion, progression and response to therapy.

PD-L1 is a type 1 transmembrane protein normally expressed on resting T-cells, B-cells, dendritic cells and macrophages as well as parenchymal cells, including vascular endothelial cells and pancreatic islet cells [28]. However, it can be expressed in various tumors, including breast cancer. The reported frequency of PD-L1 expression in BC subtypes is relatively low (10–30%) when compared to other neoplasms. Its expression differs depending on the stage or subtype of cancer. The highest PD-L1 expression is shown in TNBC followed by HER2-positive breast cancer [29]. Our results confirmed these findings and even exceeded the reported frequencies. Namely, elevated PD-L1 immunoexpression was observed in 40% of the patients in our cohort. In addition, we found that an elevated expression of PD-L1 was correlated with a higher tumor and nuclear grade but had no influence on the DFI and OS. In other words, PD-L1 overexpression is associated with important clinicopathological parameters that indicate poor outcome but without a direct correlation with survival. These findings are partially in line with some other studies. For example, Wang et al. [30] showed that an elevated expression of PD-L1 is correlated with multiple clinicopathological parameters leading to bad outcomes but also with shortened overall survival. Unlike them, Sabatier et al. [31] demonstrated that the upregulation of PD-L1 was associated with better survival and better response to chemotherapy. Therefore, the prognostic value of PD-L1 expression is controversial and still under consideration. Our next result makes this question even more interesting: a low expression or absence of PD-L1 expression was significantly associated with locoregional recurrence, another parameter of poor prognosis. One would expect that the overexpression of PD-L1, which was associated with a high tumor and nuclear grade, would be associated with locoregional recurrence as well. We might be able to understand this controversy with the help of the AR expression profile.

The expression of androgen receptor is a characteristic of a distinct molecular subset of TNBC, i.e., luminal androgen receptor (LAR). The frequency of AR expression ranges from 10 to 90% [8,9]. Our study revealed a frequency of 37% in this particular cohort. The role of AR expression as a prognostic/predictive factor in TNBC is not clear. It has been reported as a favorable prognostic factor (associated with low-grade, low-stage, low-proliferative-rate tumors), as an unfavorable prognostic factor (associated with increased lymph node metastasis, increased mortality and poor disease-free survival) and as unrelated to prognosis (summarized in [6,32]). Our results support studies that promote AR as a favorable prognostic marker [33]. Precisely, our analysis revealed that the expression of AR was negatively correlated with tumor grade but positively with the DFI. In addition to these results, the immunoexpression of AR was negatively associated with the expression of Ki67, i.e., the marker of cell proliferation, which is in line with previous findings by Astvatsaturyan et al. [32] but not with those of Rhanin et al. [34], who did not find any correlation. This result supports the idea that the expression of AR is a favorable prognostic factor [33]. At the same time, the expression of Ki67 was significantly positively associated with the expression of PD-L1 in our cohort, supporting the concept of an elevated expression of PD-L1 as an unfavorable prognostic marker.

Analyzing the immunoexpression of PD-L1 and AR simultaneously, we established a significant correlation in their expression. In other words, in the patients with an elevated expression of PD-L1, AR was also expressed and vice versa. Our result is, in some way, supported by the study of Tung et al. [35]. Guided by this result, we divided the patients into two distinct groups, i.e., those with a positive immunoexpression of both proteins and those that lacked the immunoexpression of PD-L1 and AR, and we analyzed them as two distinct entities in the context of clinicopathological parameters and survival. Our results revealed that the simultaneous immunoexpression of PD-L1 and AR was significantly associated with a high tumor and nuclear grade, which we consider to be the prominent effect of a high expression of PD-L1. Most importantly, a low or lack of immunoexpression of these genes was significantly correlated with metastatic events, which is the most unfavorable event leading to the death of patients. We think that a lack of AR expression is the kay factor for such a development. However, we cannot ignore the contribution of PD-L1, because its low expression is associated with locoregional recurrence.

Finally, we analyzed the immunoexpression of EGFR, which was shown to be very important in the stratification of TNBCs into risk groups [32]. We observed a high expression of EGFR in 34% of the analyzed patients and concluded that it was significantly correlated with metastatic events. Taking into account the previously presented and discussed results, we believe that a low PD-L1/low AR/high EGFR immunoexpression profile, in support with a high expression of Ki-67, represents a high-risk profile for TNBC patients. We think that these genes should be considered as a single package of genes and should be analyzed simultaneously, not individually.

PD-L1 overexpression can activate the PI3K/AKT/mTOR pathway, while PI3K/AKT/mTOR can regulate PD-L1 expression [36]. Therefore, we think that it is reasonable to link our previous findings [37] related to the PI3K/AKT/mTOR pathway with the present ones. Merging them, we obtain two packages of genes that should be analyzed simultaneously and could give us a real “high-risk” profile for TNBC patients: a reduced PTEN/high PI3K/high mTOR immunoexpression and low PD-L1/low AR/high EGFR immunoexpression profile.

If we take into account that the PI3K/AKT/mTOR pathway may participate in the regulation of PD-L1 expression and that abnormal PI3K/AKT/mTOR pathway activation results in increased PD-L1 protein translation, whereas PD-L1 overexpression can activate the PI3K/AKT/mTOR pathway inversely, we can bring together our previous findings with the present ones. Namely, our previous findings on another package of genes showed reduced PTEN/high PI3K/high mTOR expression as a “high-risk” TNBC profile [37]. Merging them, we obtain two packages of genes that should be analyzed simultaneously and could give us a real “high-risk” profile for TNBC patients: a reduced PTEN/high PI3K/high mTOR immunoexpression and low PD-L1/low AR/high EGFR immunoexpression profile.

## 5. Conclusions

According to our results, when we look at the impact of expression of PD-L1, AR and EGFR in the TNBC patients individually, we can come to a conclusion that the overexpression of PD-L1 and EGFR is bad news, while the expression of AR is beneficial. These results are supported by the expression of Ki-67, which was correlated with a high expression of PD-L1 and EGFR and a low expression of AR. However, PD-L1 and AR displayed a synchronized expression, i.e., they were simultaneously over- or down-expressed. When we analyzed them as a package, the conclusion was a bit different; a low expression of PD-L1 and a lack of expression of AR displayed a really bad development for these patients. Adding EGFR expression to this result brought us to the conclusion that low PD-L1/low AR/high EGFR immunoexpression, with the support of Ki-67 expression, is a real “high-risk” profile for TNBC patients. Therefore, we think that these genes should be considered as a package of genes and should be analyzed simultaneously, not individually. In addition, our previous findings identified another package of genes important for TNBC development: reduced PTEN/high PI3K/high mTOR immunoexpression. We believe that these two packages could be a promising formula for the better prediction and exploration of better treatment modalities for TNBC patients.

## Figures and Tables

**Table 1:** TNBC patients’ characteristics.

Parameters	np (%)
Age at diagnosis	
<50	99 (79.2)
≥50	26 (20.8)
Tumor type	
Ductal	79 (63.2)
Lobular	16 (12.8)
Mixed (ductal/lobular)	11 (8.8)
Other *	19 (15.2)
Tumor size (cm)	
≤2	38 (30.4)
2 -5	76 (60.8)
≥5	11 (8.8)
Tumor grade	
G2	58 (46.4)
G3	67 (53.6)
Nuclear grade	
NG1	5 (4)
NG2	51 (40.8)
NG3	69 (55.2)
Lymph node status	
Negative	79 (63.2)
Positive	45 (36)
Unknown	1 (0.8)
Locoregional reccurance	
No	112 (89.6)
Yes	13 (10.4)
Distant metastases	
No	96 (76.8)
Yes	29 (23.2)

Abbreviations: (np) number of patients per group; (*) medullary, tubular and other rare breast carcinoma types.

**Table 2:** Association between PD-L1, AR and EGFR immunoexpression and clinicopathological parameters of TNBC.

	PD-L1			AR			EGFR		
	np (%)			np (%)			np (%)		
**Parameters**	Negative	Positive	*p* value	Negative	Positive	*p* value	Negative	Positive	*p* value
	75 (60)	50 (40)		79 (63)	46 (37)		83 (66)	42 (34)	
**Tumor size**									
≤2 cm	23 (30)	15 (30)	0.6417	21 (26)	17 (37)	0.4657	27 (33)	11 (24)	0.5844
2 -5 cm	44 (59)	32 (64)		51 (65)	25 (54)		50 (60)	26 (62)	
>5 cm	8 (11)	3 (6)		7 (9)	4 (9)		6 (7)	5 (14)	
**Tumor grade**									
G2	45 (60)	13 (26)	**0.0002**	31 (39)	27 (59)	**0.0419**	36 (43)	22 (52)	0.3505
G3	30 (40)	37 (74)		48 (61)	19 (41)		47 (57)	20 (48)	
**Nuclear grade**									
NG1	4 (5)	1 (2)	**0.0007**	4 (5)	1 (2)	0.2425	2 (2)	3 (7)	0.3796
NG2	40 (54)	11 (22)		28 (35)	23 (50)		33 (40)	18 (43)	
NG3	31 (41)	38 (76)		47 (60)	22 (48)		48 (58)	21 (50)	
**Lymph node status**									
Negative	45 (60)	34 (69)	0.3416	51 (65)	28 (61)	0.6998	55 (67)	24 (57)	0.3255
Positive	30 (40)	15 (31)		27 (35)	18 (39)		27 (33)	18 (43)	
**N stage**									
N0	45 (60)	34 (69)	0.5684	51 (65)	28 (61)	0.4912	55 (67)	24 (57)	0.4946
N1	24 (32)	12 (25)		23 (30)	13 (28)		21 (26)	15 (36)	
N2 and N3	6 (8)	3 (6)		4 (5)	5 (11)		6 (7)	3 (7)	
**Metastases**									
No	57 (76)	39 (78)	0.8322	57 (72)	39 (85)	0.1273	69 (83)	27 (64)	**0.0249**
Yes	18 (24)	11 (22)		22 (28)	7 (15)		14 (17)	15 (36)	
**Locoreg. reccurence**									
No	63 (84)	49 (98)	**0.0146**	69 (87)	43 (93)	0.368	75 (90)	37 (88)	0.7597
Yes	12 (16)	1 (2)		10 (13)	3 (7)		8 (10)	5 (12)	
**Adjuvant radiotherapy**									
No	28 (37)	16 (33)	0.7008	27 (34)	17 (38)	0.8457	27 (34)	17 (40)	0.5524
Yes	46 (63)	32 (67)		50 (66)	28 (62)		53 (66)	25 (60)	
**Ki67**									
≤15%	14 (19)	2 (4)	**0.0148**	1 (1)	15 (32)	**<0.0001**	11 (13)	5 (12)	0.1831
16–30%	8 (11)	2 (4)		4 (5)	6 (14)		4 (5)	6 (14)	
>30%	53 (70)	46 (92)		74 (94)	25 (54)		68 (82)	31 (74)	

Abbreviations: np, number of patients per group. Bold indicates statistically significant values, *p* ≤ 0.05.

**Table 3:** Association between PD-L1 and AR simultaneous expression and clinicopathological parameters of TNBC.

	PD-L1/AR Expression		
	np (%)		
**Parameters**	Negative	Positive	*p* value
	42 (76)	13 (24)	
**Tumor size**			
≤2 cm	10 (24)	4 (31)	0.8344
2–5	27 (64)	8 (62)	
>5 cm	5 (12)	1 (7)	
**Tumor grade**			
G2	20 (48)	2 (15)	**0.0533**
G3	22 (52)	11 (85)	
**Nuclear grade**			
NG1	3 (7)	0 (0)	**0.0242**
NG2	18 (43)	1 (7)	
NG3	21 (50)	12 (93)	
**Lymph nodes status**			
Negative	28 (67)	11 (85)	0.3037
Positive	14 (33)	2 (15)	
**N stage**			
N0	28 (67)	11 (85)	0.2955
N1	12 (28)	1 (7)	
N2 and N3	2 (5)	1 (7)	
**Metastases**			
No	31 (74)	13 (100)	**0.0497**
Yes	11 (26)	0 (0)	
**Locoreg. reccurence**			
No	33 (79)	13 (100)	0.0961
Yes	9 (21)	0 (0)	
**Adjuvant radiotherapy**			
No	15 (36)	4 (33)	>0.9999
Yes	26 (64)	8 (67)	
**Ki67**			
≤15%	0 (0)	1 (7)	0.1452
16–30%	2 (5)	0 (0)	
>30%	40 (95)	12 (93)	

Abbreviations: np, number of patients per group. Bold indicates statistically significant values, *p* ≤ 0.05.

## Data Availability

The data presented in this study are available on request from the corresponding author.
